# hnRNPA1 impedes snakehead vesiculovirus replication via competitively disrupting viral phosphoprotein-nucleoprotein interaction and degrading viral phosphoprotein

**DOI:** 10.1080/21505594.2023.2196847

**Published:** 2023-04-02

**Authors:** An-Qi Liu, Xiangmou Qin, Hui Wu, Hao Feng, Yong-An Zhang, Jiagang Tu

**Affiliations:** a State Key Laboratory of Agricultural Microbiology, Hubei Hongshan Laboratory, Engineering Research Center of Green Development for Conventional Aquatic Biological Industry in the Yangtze River Economic Belt, Ministry of Education, College of Fisheries, Huazhong Agricultural University, Wuhan, China; b State Key Laboratory of Developmental Biology of Freshwater Fish, College of Life Science, Hunan Normal University, Changsha, China

**Keywords:** HnRNPA1, P protein, Snakehead vesiculovirus (SHVV), Nucleocytoplasmic shuttling, Replication

## Abstract

Heterogeneous nuclear ribonucleoprotein A1 (hnRNPA1) plays an important role in regulating the replication of many viruses. However, it remains elusive whether and how hnRNPA1 regulates fish virus replication. In this study, the effects of twelve hnRNPs on the replication of snakehead vesiculovirus (SHVV) were screened. Three hnRNPs, one of which was hnRNPA1, were identified as anti-SHVV factors. Further verification showed that knockdown of hnRNPA1 promoted, while overexpression of hnRNPA1 inhibited, SHVV replication. SHVV infection reduced the expression level of hnRNPA1 and induced the nucleocytoplasmic shuttling of hnRNPA1. Besides, we found that hnRNPA1 interacted with the viral phosphoprotein (P) via its glycine-rich domain, but not with the viral nucleoprotein (N) or large protein (L). The hnRNPA1-P interaction competitively disrupted the viral P-N interaction. Moreover, we found that overexpression of hnRNPA1 enhanced the polyubiquitination of the P protein and degraded it through proteasomal and lysosomal pathways. This study will help understanding the function of hnRNPA1 in the replication of single-stranded negative-sense RNA viruses and providing a novel antiviral target against fish rhabdoviruses.

## Introduction

Heterogeneous nuclear ribonucleoproteins (hnRNPs) are a group of host RNA-binding proteins that have been reported to promote or suppress virus replication [1]. As antiviral factors, hnRNPAB and hnRNPA2/B1 respectively interact with nucleoprotein (NP) and nonstructural protein 1 (NS1) of influenza A virus to restrict viral mRNA nuclear export, thus inhibiting viral replication [2,3]. hnRNPL interacts with the internal ribosome entry site (IRES) at the 5’ untranslated region of genome RNA of foot-and-mouth disease virus to suppress viral RNA synthesis, therefore inhibiting viral replication [4]. As pro-viral factors, hnRNPA2/B1 and hnRNPF respectively interact with NP and NS1 of influenza A virus to promote viral replication [5,6]. hnRNPH1 interacts with the V protein of Newcastle disease virus and promotes viral replication [7]. In addition, hnRNPA2 interacts with the core protein and nonstructural protein 5 of Japanese encephalitis virus to enhance the viral replication [8]. So far, more than twenty kinds of hnRNPs have been identified, the most abundant member of which is heterogeneous nuclear ribonucleoprotein A1 (hnRNPA1) [9].

The host factor hnRNPA1 contains two RNA binding domains (RBDs), a glycine-rich region, and a M9 nuclear localization sequence (NLS) [10,11]. hnRNPA1 has been reported to regulate the life cycle of various viruses [10]. On the one hand, it acts as an antiviral factor. hnRNPA1 inhibits human T-cell lymphotropic virus replication via disrupting viral non-structural regulatory protein REX from binding to the viral RNAs [12]. hnRNPA1 binds with viral RNA and viral polymerase NS5B to inhibit hepatitis C virus replication [13,14]. Besides, hnRNPA1 interacts with NP of influenza A virus and inhibits viral replication [15]. On the other hand, it acts as a pro-viral factor. hnRNPA1 binds to the 5’ nontranslated region of viral RNAs to promote the replication of Sindbis virus, enterovirus 71, and human rhinovirus [16–18]. hnRNPA1 binds with the NP of porcine epidemic diarrhoea virus and promotes viral replication [19]. Besides, hnRNPA1 binds to and mediates nucleocytoplasmic shuttling of p17 protein of avian reovirus to promote viral replication [20]. However, the role of hnRNPA1 in fish virus replication has rarely been investigated.

Rhabdovirus contains a ~ 11kb negative-sense RNA, which usually encodes nucleoprotein (N), phosphoprotein (P), matrix protein (M), glycoprotein (G), and large protein (L) [21]. The multifunctional P protein is a cofactor of the polymerase L with the ability to stabilize the L protein [22,23]. Besides, it binds to N protein and facilitate the encapsidation of the viral genome and the recruitment of the viral polymerase (L) onto the RNA template [24,25]. In addition, P protein can regulate viral replication via interacting with host proteins. The P protein of Peste des petits ruminants virus promotes viral replication through interacting with interferon regulatory factor 3 (IRF3) to inhibit host antiviral innate immune response [26]. The P protein of human parainfluenza virus type 3 interacts with synaptosome-associated protein of 29 kDa (SNAP29) to inhibit the interaction between SNAP29 and syntaxin 17, thus facilitating virus production [27]. The rabies virus (RV) P protein interacts with the ATP binding cassette E1 (ABCE1) to promote viral replication via facilitating P-mediated disruption of host antiviral immune response, and the RV P also binds to the ribosomal protein L9 in the cytoplasm to inhibit the virus transcription, thus suppressing viral replication [28,29].

Snakehead vesiculovirus (SHVV), isolated from diseased hybrid snakehead fish, is a kind of single-strand negative-sense RNA virus belong to *rhabdoviridae* family [30]. In this study, the effects of twelve hnRNPs on SHVV replication were screened, and hnRNPA1 was selected for further study as it is the most abundant member in hnRNPs family and plays important roles in virus replication. SHVV infection reduced the mRNA and protein levels of hnRNPA1 and induced the nucleocytoplasmic shuttling of hnRNPA1. hnRNPA1 was identified as an anti-SHVV factor with the mechanisms that hnRNPA1 disrupted the viral P-N interaction via competitively binding with and degrading the viral P protein through proteasomal and lysosomal pathways. Our study provides information on the role of hnRNPA1 in fish virus replication, which helps designing antiviral drugs for fish rhabdoviruses.

## Materials and methods

### Cells and viruses

293T cells were cultured in Dulbecco’s modified eagle medium (DMEM) (Gibco) containing 10% foetal bovine serum (FBS) (Gibco) and 100 g/ml penicillin-streptomycin (Gibco) at 37°C and 5% CO_2_ atmosphere. Channel catfish ovary (CCO) cells were cultured in minimum essential medium (MEM) (Gibco) containing 10% FBS (Gibco) and 100 mg/ml penicillin-streptomycin (Gibco) at 25°C. SHVV (GenBank No. KP876483.1) was stored at −80°C in our laboratory.

### Plasmids

The plasmids pHA-Ub, pMyc-P, pHis-P, pHis-N, and pCDNA-L have been constructed previously [31]. The plasmid pFlag-hnRNPA1 was constructed by amplifying the *hnRNPA1* gene from CCO cells, followed by cloning into p3×Flag-CMV-14. The plasmids expressing EGFP-fused RRM region, glycine-rich region, or M9 region of hnRNPA1 were constructed by amplifying hnRNPA1 segments from pFlag-hnRNPA1 and cloning into pEGFP-N1. All the primers are listed in Table 1.

**Table 1.** Primers used in this study.

Application	Primer	Sequence (5′–3′)
Expression	Flag-hnRNPA1-F	GAACCGTCAGAATTAAGCTTATGCCAAAAGAGGATCACCC
	Flag-hnRNPA1-R	TCAGATCTATCGATGAATTCGCATATCGTCTACCACCAGAGCCTC
	EGFP-hnRNPA1RRM-F	CCCAAGCTTATGCCAAAAGAGGATCACCC
	EGFP-hnRNPA1RRM-R	GGGGTACCGTCTCTTGTTTAGAGAGGGCCT
	EGFP-hnRNPA1Glyrich-F	CCCAAGCTTATGCAGAATTCGTCAATGAACA
	EGFP-hnRNPA1Glyrich-R	GGGGTACCGTGCCACCACTGCCACCACCACCACCT
	EGFP-hnRNPA1M9-F	CCCAAGCTTATGAGCTACAGTGACTTTGGAAAC
	EGFP-hnRNPA1M9-R	GGGGTACCGTATATCGTCTACCACCAGAGC
qRT-PCR	q-G-F	ACACCATACATGCCAGAGGC
	q-G-R	GCCTCGCTGGGTATCCAAAT
	q-P-F	TGTGCTCTGGGCTTCTGG
	q-P-R	TTTCCGGCTGGGAGTTTT
	q-*β*-actin-F	AGCCATCCTTCTTGGGTATG
	q-*β*-actin-R	GGTGGGGCGATGATCTTGAT
	q-hnRNPA0-F	AAGCGGAAGTCATCACGG
	q-hnRNPA0-R	CAGCCTGTATTTCTTGTTTAGT
	q-hnRNPA0a-F	AGGCTGTAGTGCTCAAGTTTC
	q-hnRNPA0a-R	CATACCCGCCGTCGTTTC
	q-hnRNPA1-F	TGGGTGGCATCAAGGAAG
	q-hnRNPA1-R	TTATGGCCGTTTATCGTG
	q-hnRNPA1a-F	ATGTCTAAAGAGACGCCTCGCGAGC
	q-hnRNPA1a-R	GCTCGCGAGGCGTCTCTTTAGACAT
	q-hnRNPA3-F	AGCGGTATGGGAAGATTG
	q-hnRNPA3-R	CTTCACAGTTATGGGAGTTT
	q-hnRNPD0-F	ACGAGGAAGATGAAGGGAA
	q-hnRNPD0-R	AAACAGCACGAAGCCAAA
	q-hnRNPDlike-F	ATGTGGGAGCAGGAAAGT
	q-hnRNPDlike-R	TGTAGCCATTGCCGTAGC
	q-hnRNPH-F	GGGACACCGTTATGTAGAAG
	q-hnRNPH-R	AAGCAAACTGCACGAAGG
	q-hnRNPH3-F	TAGCCCATACGACAGACC
	q-hnRNPH3-R	TGACCTCCCATTGTTCCT
	q-hnRNPH1like-F	TCTCAGGGTTGGAAATCG
	q-hnRNPH1like-R	TCAGCACGGCTGCTCTTG
	q-hnRNPK-F	TCCTGGGATAACTATCATTCTG
	q-hnRNPK-R	AATCTGGTCCTGCGTGCC
	q-hnRNPKlike-F	ACAATGCCAGTGTATCAGTC
	q-hnRNPKlike-R	ATTCTTTAATCTTGGTCCCT
	q-hnRNPL-F	GACCAGGACACTTGGGATT
	q-hnRNPL-R	CGTATGTCTCATCGTAGGGA
	q-hnRNPL2-F	GAGTTATGGCGGGTATGA
	q-hnRNPL2-R	CCTGTCCGCATTTATTTT
	q-hnRNPLlike-F	GAGATGGGCGACGAGTAT
	q-hnRNPLlike-R	TGAAGGAGGTTGGATGATGT
	q-hnRNPM-F	GAGGCGGTAGATACGAGC
	q-hnRNPM-R	CACAGCCTTCTTCATCAGTT
Application	Primer	Sequence (5′–3′)
	q-hnRNPQ-F	AGGCACTCAGCCCTCCAT
	q-hnRNPQ-R	CCTCGGTTCAGACCACTCA
	q-hnRNPR-F	CTAAGCCTCCAGATAAGAAGA
	q-hnRNPR-R	AGTAATCGGGTGGGTAAGA
	q-hnRNPU1like-F	ACACCTATAACTGCGACCTG
	q-hnRNPU1like-R	ACCCGATTCTCACCACAT
	q-hnRNPU2like-F	ACTGTGAGGTGCTGATGTT
	q-hnRNPU2like-R	TTGGTTGCTGCTATTCTG
	q-hnRNPUb-F	ACCTTGGCGTGGCATTCA
	q-hnRNPUb-R	CTCGCAGTCTTTCTTGGTCTCA

### Reagents and antibodies

NH_4_Cl (E0151), MG132 (S2619), and 3-MA (S2767) were obtained from Selleck Biotechnology Co., LTD. (Shanghai, China). Small interfering RNAs (siRNAs) of hnRNPs were obtained from GenePharma Co., LTD. (Suzhou, China). The rabbit anti-SHVV-N, -SHVV-P, -SHVV-L, and -hnRNPA1 polyclonal antibodies were obtained from Abiotech (Jinan, China). The mouse anti-*β*-actin (AC004), anti-EGFP (AE012), anti-Flag (AE005), anti-Myc (AE010), and anti-His (AE003) and rabbit anti-Histone H3 (A2348) antibodies were obtained from ABclonal Biotechnology Co., LTD. (Wuhan, China). The HRP-conjugated goat anti-rabbit IgG (AS014) and anti-mouse IgG (AS003) antibodies were obtained from ABclonal Biotechnology Co., LTD. (Wuhan, China).

### Virus infection and titration

CCO cells were incubated with SHVV at a multiplicity of infection (MOI) of 0.1 for 2 h, and the medium was replaced by MEM with 2% FBS. At different time points post of SHVV infection, the cells were collected for the detection of mRNA and protein levels of hnRNPA1 and viral genes by quantitative reverse transcription-PCR (qRT-PCR) and Western blotting, while the supernatants were detected for virus titre by 50% tissue culture infectious dose (TCID_50_).

### Transfection

Transfections were performed as previously described [32]. Briefly, plasmid was mixed with TransIntro^TM^ EL transfection reagent (TransGen Biotech, FT231–02) in Opti-MEM medium (Invitrogen, 31985070) at room temperature for 20 min. The mixed solution is then placed onto the cell plates. After 6 h incubation, MEM or DMEM with 10% FBS was used to replace the Opti-MEM medium, and the cells were cultured further for 18 h.

### siRNA knockdown

The siRNAs targeting hnRNPs were synthesized by GenePharma (Suzhou, China) based on the sequences of channel catfish hnRNPs. All the sequences of the siRNAs were listed in Table 2. The siRNA knockdowns of the hnRNPs were performed as previously described [33]. Briefly, siRNAs were transfected into CCO cells using TransIntro^TM^ EL transfection reagent. The samples were examined by Western blotting or qRT-PCR at 24 h post of transfection. As the most abundant member in hnRNP family [10], hnRNPA1 was chosen for further study.

**Table 2.** siRnas used in this study.

Gene	Gene ID	Sequence	
		sense(5”−3”)	antisense(5”−3”)
hnRNPA0a	108269032	CCUGACCAAGCAGGAGAUGTT	CAUCUCCUGCUUGGUCAGGTT
hnRNPA1	108275986	GGCCUCAGCUUUGAGACAATT	UUGUCUCAAAGCUGAGGCCTT
hnRNPA3	108266578	CUGCUUUGUAACGUUUGAUTT	AUCAAACGUUACAAAGCAGTT
hnRNPH	108269107	GGCUGCAGCAAAGAGGAGATT	UCUCCUCUUUGCUGCAGCCTT
hnRNPH3	108263529	CAACUACUGCUUCGGUAAUTT	AUUACCGAAGCAGUAGUUGTT
hnRNPM	108271081	ACCCGCUCCAGGGCUCUGATT	UCAGAGCCCUGGAGCGGGUTT
hnRNPQ	108255093	CUACUCAGCGCUACGGCAGTT	CUGCCGUAGCGCUGAGUAGTT
hnRNPR	108275175	CCUAUGAUGUCAAAUGGCATT	UGCCAUUUGACAUCAUAGGTT
hnRNPUb	108257736	ACCUGCGGCUGAUGAUGGATT	UCCAUCAUCAGCCGCAGGUTT
hnRNPDlike	108276833	AGAAGGAGACGCCGUAUUUTT	AAAUACGGCGUCUCCUUCUTT
hnRNPH1like	100304950	CACAGGGUAUGAUGAUUAUTT	AUAAUCAUCAUACCCUGUGTT
hnRNPUlike2	108275330	GACCAAGUCAGGGCUACAATT	UUGUAGCCCUGACUUGGUCTT

### QRT-PCR

Total RNA was extracted from CCO cells with TRIzol reagent (Takara, 9109). To detect the mRNA levels of SHVV-G, SHVV-P, or hnRNPs, 1 µg RNA was reverse transcribed to cDNA according to the manufacturer’s instructions of PrimeScript^TM^RT reagent Kit (Takara, RR037A). Then, 2 µl cDNA template, 0.5 µl forward primer, 0.5 µl backward primer, 7 µl ddH_2_O, and 10 µl AceQ qPCR SYBR Green Master mix (Vazyme, Q131–02) were mixed for the quantitative PCR reactions. The reactions were based on the following cycles: ①95°C for 5 min; ②45 cycles at 95°C for 10 s, 60°C for 10 s, and 72°C for 15 s; ③95°C with 5°C/s calefactive velocity. Data were normalized to the level of *β*-actin and analysed using the 2^−ΔΔCt^ method.

### Immunofluorescence (IF) assay

Immunofluorescence (IF) assays were performed as previously described [34]. Briefly, at different time points post of SHVV infection, CCO cells on glass coverslips were fixed with 4% paraformaldehyde for 15 min at room temperature, and treated with 0.5% Triton X-100 for 15 min, followed by blocking with 5% bovine serum albumin (BSA) for 1 h. After three-time washing with PBS, the cells were incubated with primary antibody (1:100) for 4 h and fluorescein isothiocyanate (FITC)-conjugated goat anti-rabbit IgG antibody (1:200) for 1 h, followed by incubation with 10 µg/mL 4′, 6-diamidino-2-phenylindole (DAPI) for 10 min. After washing with PBS for three times, the samples were imaged using confocal microscope (Nikon N-STORM).

### Western blotting

The protein samples were separated by sodium dodecyl sulphate polyacrylamide gel electrophoresis (SDS-PAGE), followed by transferring onto nitrocellulose filter membrane (Biosharp, 20030). The membranes were blocked with 5% skim milk in tris-buffered saline with 0.1% tween 20 (TBST) for 2 h and then incubated with the anti-N (1:1000), anti-P (1:1000), anti-M (1:1000), anti-G (1:1000), anti-L (1:1000), anti-hnRNPA1 (1:1000), anti-Flag (1:2000), anti-Myc (1:4000), anti-HA (1:4000), anti-EGFP (1:2000), anti-Histone H3 (1:4000), or anti-*β*-actin (1:5000) antibodies for 4 h. After three-time washing with TBST, the membranes were incubated with HRP-conjugated goat anti-rabbit antibody (1:5000) or anti-mouse antibody (1:5000) for 1 h. The signal intensity was visualized using Amersham Imager 600 System after washing with TBST for three times.

### Co-Immunoprecipitation (Co-IP)

Cells were lysed in Pierce™ IP lysis buffer (Thermo Scientific, 87787). The immunoprecipitation was conducted using anti-Flag, anti-Myc or anti-EGFP antibody according to the manufacturer’s instructions of protein A/G (MedChemExpress, HY-K0202). The eluted samples were then boiled with 2×SDS buffer for 10 min, followed by Western blotting.

### Nuclear and cytoplasmic fractionation

SHVV-infected or Mock- cells were collected, and the nuclear and cytoplasmic fractions were fractionated using the Nuclear and Cytoplasmic Protein Extraction Kit (Beyotime, P0027) according to the manufacturer’s instructions. Histone-H3 was used as nuclear internal control, while *β*-actin was used as cytoplasmic internal control for Western blotting.

### Statistical analysis

Graphpad Prism (version 6.0; La Jolla, CA, USA) was used to analyse the data. Student’s *t*-test was used to determine the statistical significance of the data. The * and ** indicate statistically significant differences (*, *p* < 0.05; **, *p* < 0.01).

## Results

### Screening the effects of hnRnps on SHVV replication

**Figure 1.**
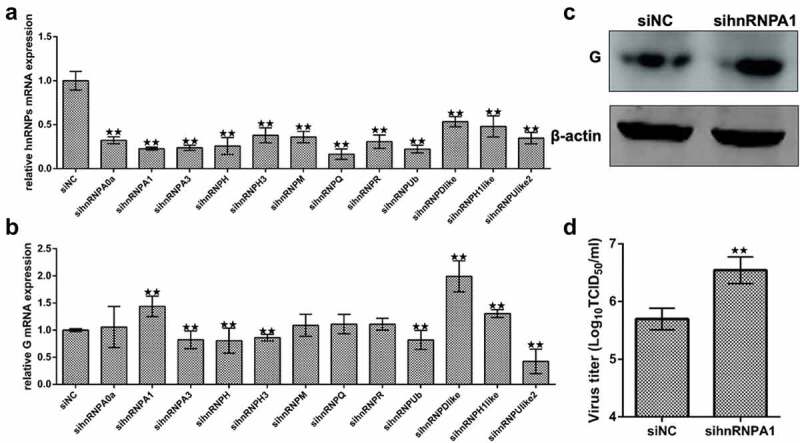
Screening the effects of hnRnps on SHVV replication. (A) CCO cells were transfected with siRnas targeting each of the twelve hnRnps, and the total RNAs were extracted at 24 h post transfection. The mRNA level of the hnRnps was measured using qRT-PCR, *β-actin* was used as the internal control. (B) CCO cells were transfected with siRnas targeting each of the twelve hnRnps, followed by SHVV infection. The total RNAs were extracted at 24 h post of SHVV infection, and the viral G mRNA levels in cells were measured using qRT-PCR, *β-actin* was used as the internal control. (C-D) CCO cells were transfected with siNC or sihnRNPA1, followed by SHVV infection. The G protein levels in cells were measured using Western blotting with β-actin as the internal control, while the viral titre in supernatants was measured using TCID_50_. All the data are performed in triplicate (mean ± sd). The * and ** indicate statistically significant differences (*p < 0.05; **p < 0.01).

To screen the effects of hnRNPs on SHVV replication, siRNA knockdown of the hnRNPs was performed (Figure 1A and B). The results indicated that knockdown of hnRNPA1, heterogeneous nuclear ribonucleoprotein D-like (hnRNPDlike), or heterogeneous nuclear ribonucleoprotein H1-like (hnRNPH1like) increased viral G mRNA levels, while knockdown of heterogeneous nuclear ribonucleoprotein A3 (hnRNPA3), heterogeneous nuclear ribonucleoprotein H (hnRNPH), heterogeneous nuclear ribonucleoprotein H3 (hnRNPH3), heterogeneous nuclear ribonucleoprotein Ub (hnRNPUb), or heterogeneous nuclear ribonucleoprotein U-like2 (hnRNPUlike2) decreased viral G mRNA levels (Figure 1B), indicating that hnRNPA1, hnRNPDlike, and hnRNPH1like were anti-SHVV factors, while hnRNPA3, hnRNPH, hnRNPH3, hnRNPUb, and hnRNPUlike2 were pro-SHVV factors.

To detect the levels of viral G protein and viral titre, Western blotting and TCID_50_ were performed. The results showed that the levels of viral G protein and viral titre were increased when hnRNPA1 was knocked down (Figure 1C and D), suggesting that knockdown of hnRNPA1 promoted SHVV replication.

### Overexpression of hnRNPA1 inhibits SHVV replication

**Figure 2.**
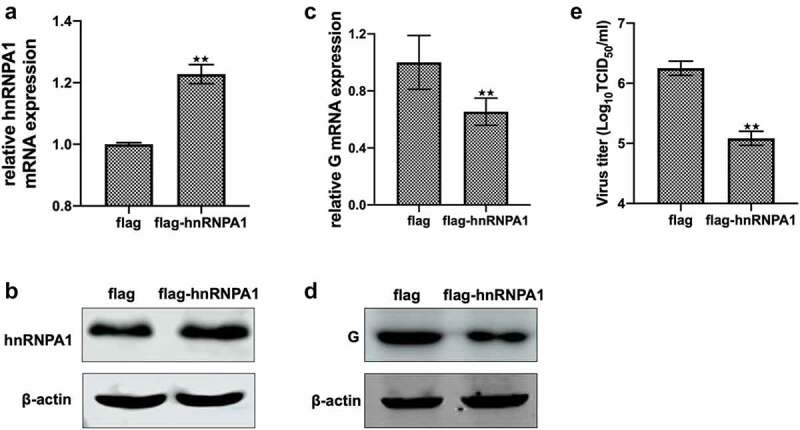
Effect of overexpression of hnRNPA1 on SHVV replication. (A and B) CCO cells were transfected with pFlag-hnRNPA1 or p3×flag-CMV-14 (control). The mRNA and protein levels of hnRNPA1 were measured using qRT-PCR and Western blotting, and β-actin was used as the internal control. (C-E) CCO cells were transfected with pFlag-hnRNPA1 or p3×flag-CMV-14, followed by SHVV infection. The viral G mRNA and protein levels in cells were measured using qRT-PCR and Western blotting, and β-actin was used as the internal control, while the viral titre in supernatants was measured using TCID_50_. All the data are performed in triplicate (mean ± sd). The * and ** indicate statistically significant differences (*p < 0.05; **p < 0.01).

Overexpression of hnRNPA1 was confirmed by detecting the mRNA and protein levels of hnRNPA1 (Figure 2A and B). The levels of G mRNA, G protein, and viral titre were decreased when hnRNPA1 was overexpressed (Figure 2C-E), suggesting that overexpression of hnRNPA1 inhibited SHVV replication. All these results suggest that hnRNPA1 is an anti-SHVV factor.

### SHVV infection reduces hnRNPA1 expression

**Figure 3.**
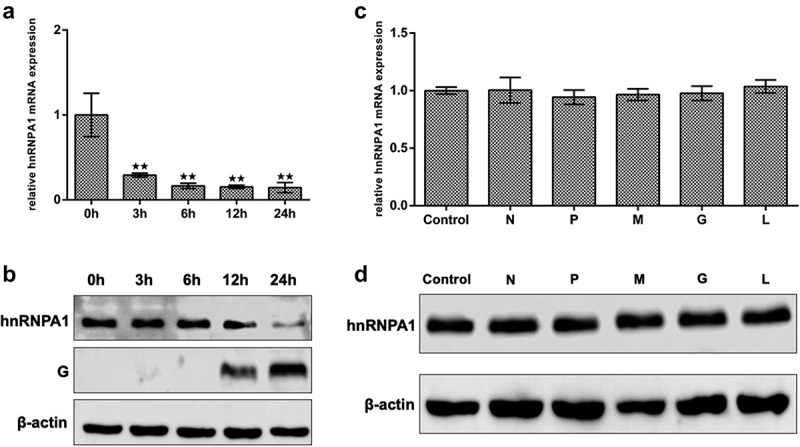
Expression of hnRNPA1 during SHVV infection. (A) CCO cells were infected with SHVV, and the cells were harvested at 0, 3, 6, 12, and 24 h. The mRNA levels of hnRNPA1 were measured using qRT-PCR, and *β-actin* was used as the internal control. (B) the protein levels of hnRNPA1 and SHVV G were measured using Western blotting, and β-actin was used as the internal control. (C-D) the plasmids expressing N, P, M, G, or L were transfected into CCO cells, the empty vector pCDNA3.1 was used as control. The mRNA and protein levels of hnRNPA1 were measured using qRT-PCR, and β-actin was used as the internal control. All the data are performed in triplicate (mean ± sd). The * and ** indicate statistically significant differences (*p < 0.05; **p < 0.01).

To determine the effects of SHVV infection on the expression of hnRNPA1, qRT-PCR and Western blotting were performed. The results indicated that the mRNA and protein levels of hnRNPA1 were decreased during SHVV infection (Figure 3A and B). To evaluate the viral proteins responsible for the reduction of hnRNPA1 expression, five plasmids expressing each SHVV proteins were transfected, which have been constructed previously [35] and verified to be successfully expressed (Supplementary Fig. S1). The results showed that the mRNA and protein levels of hnRNPA1 at 24 h post of transfection were not significantly affected by any of the viral proteins (Figure 3C and D), indicating that the reduction of hnRNPA1 expression caused by SHVV infection was not due to the viral proteins.

### SHVV infection induces the nucleocytoplasmic shuttling of hnRNPA1

**Figure 4.**
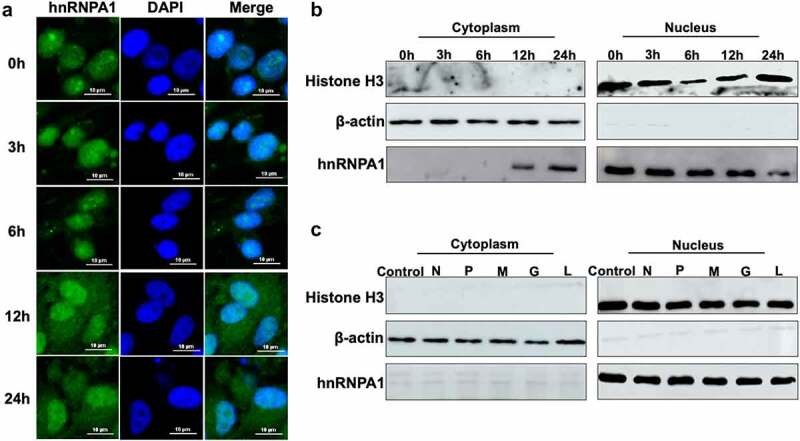
Subcellular localization of hnRNPA1 during SHVV infection. (A) CCO cells were infected with SHVV, and the cells were harvested at 0, 3, 6, 12, and 24 h post infection. The localization of hnRNPA1 was measured by immunofluorescence assay. (B) CCO cells were infected with SHVV, and the cytoplasmic and nuclear components were extracted at 0, 3, 6, 12, and 24 h post infection. Samples were detected by Western blotting with anti-hnRNPA1, anti-histone H3, and anti-β-actin antibodies. (C) the plasmids expressing N, P, M, G, or L were transfected into CCO cells, with the empty vector pCDNA3.1 used as control. The cytoplasmic and nuclear components were extracted, and the samples were detected by Western blotting with antibodies against hnRNPA1, histone H3, and β-actin.

The subcellular localization of hnRNPA1 was detected, and the results indicated that hnRNPA1 mainly located in nucleus at 0, 3, and 6 h, but partially shuttled from nucleus to cytoplasm at 12 and 24 h post of SHVV infection (Figure 4A). The hnRNPA1 in cytoplasmic and nuclear components were detected, and the results proved the nucleocytoplasmic shuttling of hnRNPA1 during SHVV infection (Figure 4B). Then, we assessed the viral proteins responsible for the nucleocytoplasmic shuttling of hnRNPA1, and our results indicated that the nucleocytoplasmic shuttling of hnRNPA1 was not caused by any of the viral proteins (Figure 4C).

### hnRNPA1 interacts with SHVV P through its Gly-rich region

**Figure 5.**
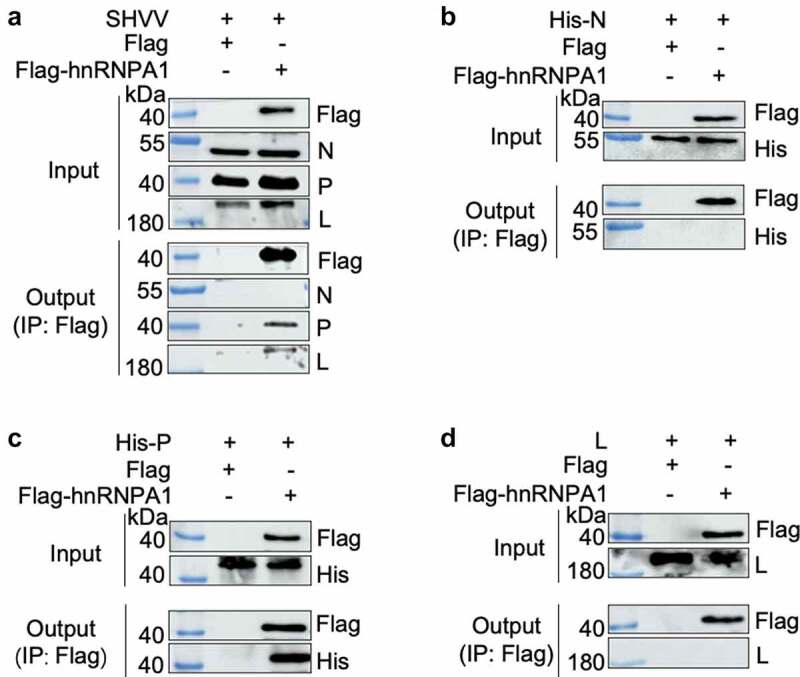
hnRNPA1 interacts with SHVV P protein. (A) CCO cells were transfected with pFlag-hnRNPA1 or p3×flag-CMV-14 (control), followed by SHVV infection. The whole-cell lysates were obtained at 24 h post infection and immunoprecipitated with anti-Flag antibody. The anti-N, anti-P, anti-L, and anti-Flag antibodies were used for Western blotting. (B-D) 293T cells were transfected with pFlag-hnRNPA1, together with the plasmids expressing His-N, His-P, or L. The whole-cell lysates were obtained at 24 h post transfection and immunoprecipitated with anti-Flag antibody. The anti-L, anti-His, and anti-Flag antibodies were used for Western blotting.

**Figure 6.**
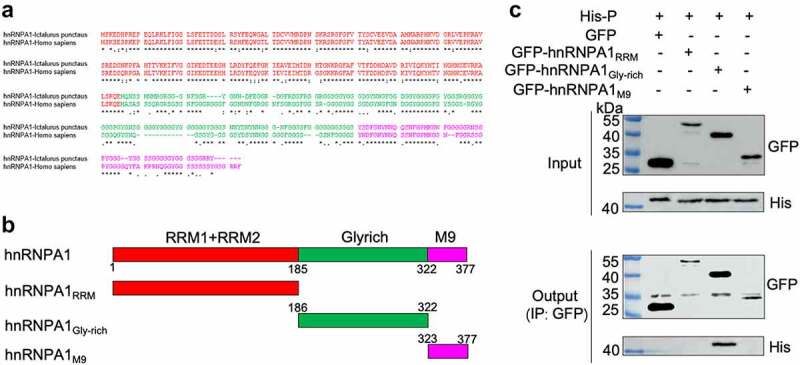
hnRNPA1 interacts with SHVV P through its Gly-rich region. (A) Sequence of hnRNPA1 from *Ictalurus punctaus* was compared with hnRNPA1 from *Homo sapiens*. Regions of hnRNPA1 was marked with different colours. (B) a series of hnRNPA1 segment plasmids expressing EGFP-fused RRM region, Gly-rich region, or M9 region were constructed. (C) 293T cells were transfected with plasmid pHis-P, together with plasmids expressing EGFP-fused RRM region, glycine-rich region, or M9 region of hnRNPA1. The whole-cell lysates were obtained at 24 h post transfection and immunoprecipitated with anti-EGFP antibody. The anti-P and anti-EGFP antibodies were used for Western blotting.

To determine the viral proteins interacting with hnRNPA1, Co-IP was performed, and the results indicated that hnRNPA1 interacted with the P and L proteins, but not the N protein (Figure 5A). Further verification using expression plasmids showed that hnRNPA1 interacted with the P protein, but not the N or L protein (Figure 5B-D), indicating that SHVV P was an interacting partner of hnRNPA1. To determine the region of hnRNPA1 responsible for the interaction, sequences of hnRNPA1 from channel catfish (*Ictalurus punctatus*) and humans (*Homo sapiens*) were aligned. The results showed that the two hnRNPA1s shared high sequence similarity (80.00%) (Figure 6A). The plasmids expressing different regions of hnRNPA1 were constructed (Figure 6B), and the Gly-rich region of hnRNPA1, but not the RRM or M9 region, interacted with SHVV P (Figure 6C).

### hnRNPA1 competitively disrupts the P-N interaction

**Figure 7.**
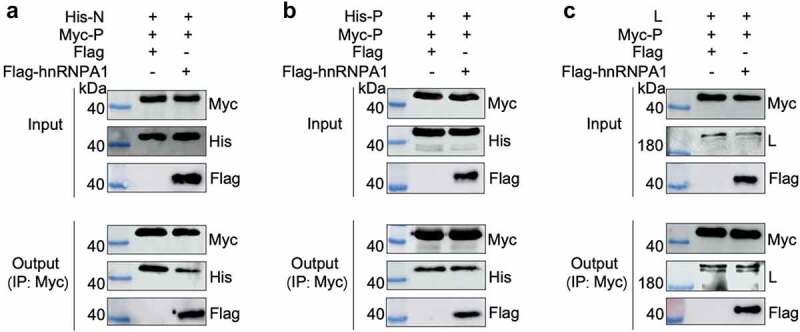
hnRNPA1 disrupts the viral P-N interaction. (A-C) 293T cells were transfected with pMyc-P and pFlag-hnRNPA1 or p3×flag-CMV-14 (control), together with plasmids expressing His-N, His-P, or L. The whole-cell lysates were obtained at 24 h post transfection and were immunoprecipitated with anti-Myc antibody. The anti-Myc, anti-His, anti-Flag and anti-L antibodies were used for Western blotting.

To explore the effect of hnRNPA1-P interaction on the interaction of the viral P-N, P-P, or P-L, Co-IP results showed that hnRNPA1 reduced the interaction between the P and N, but not the interaction of P-P or P-L (Figure 7). These results indicate that hnRNPA1 disrupts the P-N interaction via competitively binding to the P protein.

### hnRNPA1 degrades the P protein through proteasomal and lysosomal pathways

**Figure 8.**
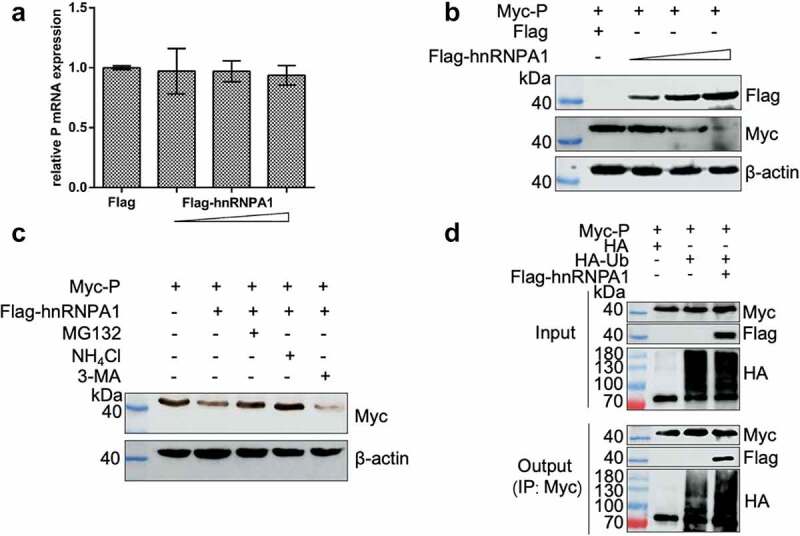
hnRNPA1 degrades SHVV P through proteasomal and lysosomal pathways. (A-B) 293T cells were transfected with pMyc-P, together with p3×flag-CMV-14 (control) or different doses of pFlag-hnRNPA1. The mRNA and protein levels of hnRNPA1 were measured using qRT-PCR and Western blotting with β-actin as the internal control. (C) 293T cells were transfected with pMyc-P together with pFlag-hnRNPA1, and the cells were treated with MG132 (5 µm), NH_4_Cl (15 µm), or 3-MA (60 µm). The cells were harvested at 24 h post transfection. The hnRNPA1 protein was determined by Western blotting, β-actin was used as the internal control. (D) 293T cells were cotransfected with pMyc-P and pHA-Ub, together with or without pFlag-hnRNPA1. The cells were collected at 24 h post transfection, and Co-IP assay was carried out with anti-Myc antibody. The anti-Myc, anti-Flag, and anti-HA antibodies were used for Western blotting.

We explored whether hnRNPA1 affects the stability of SHVV P, and the results showed that hnRNPA1 reduced the level of P protein, but not the level of P mRNA (Figure 8A and B), suggesting that hnRNPA1 degraded SHVV P. 293T cells were then co-expressed with Myc-P and Flag-hnRNPA1, followed by treatment with the proteasome inhibitor MG132, the lysosome inhibitor NH_4_Cl, or the autophagy inhibitor 3-MA. These results showed that the hnRNPA1-mediated degradation of SHVV P could be restored by MG132 and NH_4_Cl, indicating that hnRNPA1 degraded the P protein through proteasomal and lysosomal pathways (Figure 8C). To determine the effect on the polyubiquitination of SHVV P, Co-IP results showed that overexpression of hnRNPA1 enhanced the polyubiquitination of the P protein (Figure 8D).

## Discussion

In our study, we screened the effects of 12 hnRNPs on SHVV replication and identified several antiviral hnRNPs and several pro-viral hnRNPs, indicating that hnRNPs play important roles in SHVV replication, in which we chose hnRNPA1 for further study. As the most abundant member in the hnRNPs family, hnRNPA1 plays important regulatory role in the life cycle of various viruses [10]. At one aspect, it plays an antiviral role in the replication of human T-cell lymphotropic virus, hepatitis C virus, and influenza A virus [12–15]. At another aspect, it plays a pro-viral role in the replication of Sindbis virus, epidemic porcine diarrhoea virus, human rhinovirus, enterovirus 71, and avian reovirus [16–20]. However, for human immunodeficiency virus, Monette et al. found that hnRNPA1 promoted viral replication, while Zahler et al. found that hnRNPA1 inhibited viral replication [36,37]. Therefore, the role of hnRNPA1 in the replication of different viruses was complicated. Until now, the role of hnRNPA1 in fish virus replication has never been investigated. In this study, the effects of overexpression and knockdown of hnRNPA1 on SHVV replication were conducted. Our results indicated that hnRNPA1 was an anti-SHVV factor. To our knowledge, this is the first report on investigating the role of hnRNPA1 in fish virus replication.

During viral infection, host may exhibit different antiviral strategies against viral infection. As a host factor, hnRNPA1 responds to virus infection variously. When acting as an antiviral role, the expression level of hnRNPA1 was increased during influenza A virus infection, which is beneficial for host to suppress influenza A virus replication [15]. Meanwhile, when acting as a pro-viral factor, the expression level of hnRNPA1 was decreased during porcine epidemic diarrhoea virus infection, which is beneficial for host to suppress porcine epidemic diarrhoea virus replication [19]. In our study, we found that the mRNA and protein levels of hnRNPA1 that suppressed SHVV replication were decreased during SHVV infection, exhibiting a strategy used by SHVV for viral replication. Previous study showed that protease 3C of enterovirus 71 caused the reduction of hnRNPA1 during enterovirus 71 infection via degrading hnRNPA1 [38]. However, our results showed that the reduction of hnRNPA1 expression during SHVV infection was not caused by any of the viral proteins. As M9 region acts as a signal of nucleocytoplasmic shuttling, hnRNPA1 is a typical protein that transfers between nucleus and cytoplasm [39]. Previous study indicated that Seneca valley virus infection induced the nucleocytoplasmic shuttling of hnRNPA1 [40]. In our study, the nucleocytoplasmic shuttling of hnRNPA1 was observed during SHVV infection. However, the nucleocytoplasmic shuttling of hnRNPA1 was not induced by any of the viral proteins. As previous study indicated that enterovirus 71 infection induced the nucleocytoplasmic shuttling of hnRNPA1 via misshapen/NIKs-related kinase (MINK)/p38 mitogen-activated protein kinase (MAPK) pathway [41]. Further studies are needed to investigate the signal pathway upon which SHVV infection caused the reduction and nucleocytoplasmic shuttling of hnRNPA1.

As a host factor, hnRNPA1 plays its regulatory role in virus replication through interacting with viral proteins. hnRNPA1 has been reported to interact with the N protein of mouse hepatitis virus, porcine epidemic diarrhoea virus, Junin virus, SARS coronavirus, and influenza A virus [15,19,42–44]. However, the mechanism is unknown till now. Only for SARS coronavirus, hnRNPA1 was found to interact with the N protein to facilitate viral RNA synthesis, thus promoting viral replication [44]. Besides, hnRNPA1 interacted with the polymerase NS5B of hepatitis C virus to mediate the NS5B to proper region of viral genomic RNA to promote viral replication [14]. In this study, we found that hnRNPA1 interacted with SHVV P protein. Moreover, we determined that the Gly-rich region of hnRNPA1 interacted with SHVV P, consistent with previous report that the Gly-rich region of hnRNPA1 interacted the avian reovirus p17 protein [20]. The mechanism on how the hnRNPA1-P interaction affected SHVV replication was investigated in this study. We found that hnRNPA1-P interaction competitively disrupted the P-N interaction, which was important for SHVV replication. Moreover, we found that hnRNPA1 degraded SHVV P through the proteasomal and lysosomal pathways, and the overexpression of hnRNPA1 enhanced the polyubiquitination of P protein. Our results revealed novel insights into the mechanisms on how hnRNPA1 regulate viral replication.

Taken together, hnRNPA1 plays a vital role in the replication of SHVV infection. This study determined the mechanism by which hnRNPA1 impeded SHVV replication. HnRNPA1 interacted with SHVV P to competitively disrupt the P-N interaction and degraded SHVV P through the proteasomal and lysosomal pathways. Our study will help to develop antiviral fish breeds targeting hnRNPA1 and design antiviral targets for fish rhabdoviruses.

## Supplementary Material

Supplemental Material

## Data Availability

The data that support the findings of this study are available from the corresponding author upon reasonable request.
