# Supplementary material for: hnRNPA1 impedes snakehead vesiculovirus replication via competitively disrupting viral phosphoprotein-nucleoprotein interaction and degrading viral phosphoprotein
Source: Virulence. 2023 Apr 2;14(1):2196847. doi: 10.1080/21505594.2023.2196847 (PMC10072109; doi:10.1080/21505594.2023.2196847)
Supplement: Supplemental Material [file KVIR_A_2196847_SM1696.docx]

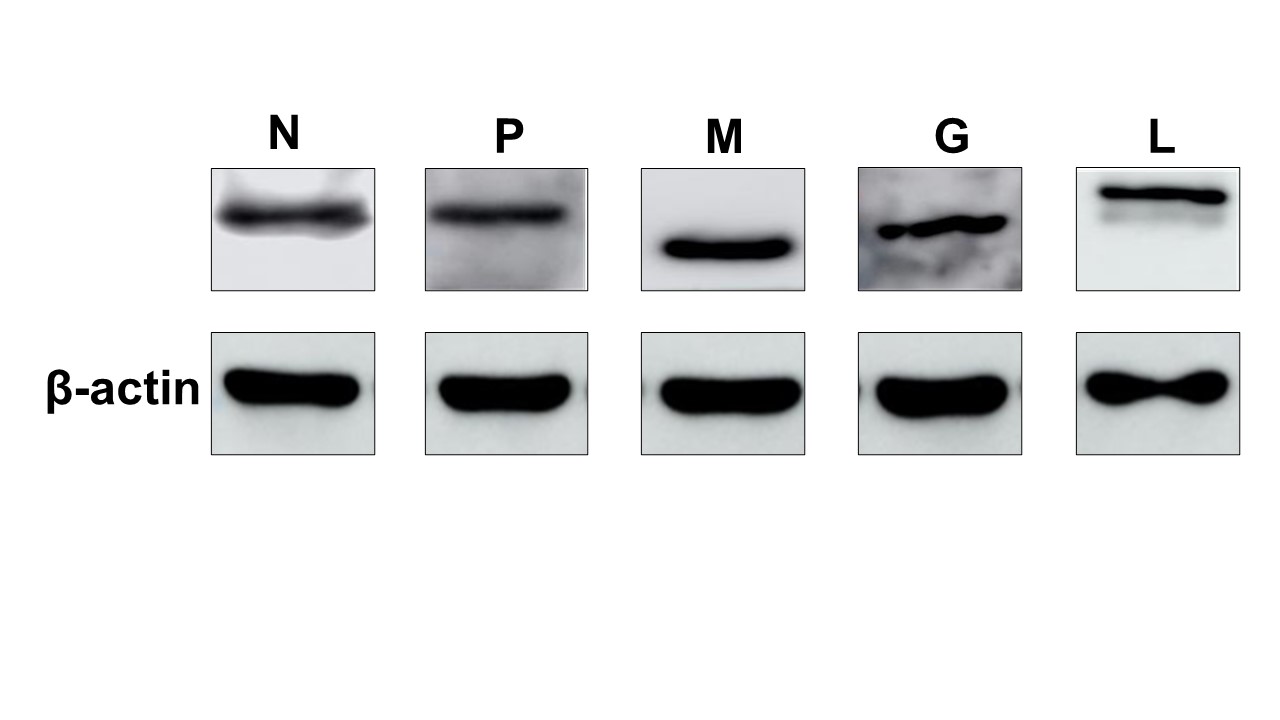


**Supplementary Figure 1. expression verification of viral proteins.** The plasmids expressing N, P, M, G, or L were transfected into 293T cells, and Western blotting was performed to detect N, P, M, G, and L protein. The anti-N, anti-P, anti-M, anti-G, and anti-L antibodies were used for Western blotting. β-actin was used as the internal control.
